# Crystal structure of TAZ-TEAD complex reveals a distinct interaction mode from that of YAP-TEAD complex

**DOI:** 10.1038/s41598-017-02219-9

**Published:** 2017-05-17

**Authors:** Hung Yi Kristal Kaan, Siew Wee Chan, Siew Kim Joyce Tan, Fusheng Guo, Chun Jye Lim, Wanjin Hong, Haiwei Song

**Affiliations:** 1 Institute of Molecular and Cell Biology, A*STAR (Agency for Science, Technology and Resesarch), 61 Biopolis Drive, Singapore, 138673 Singapore; 2 Department of Biochemistry, National University of Singapore, 14 Science Drive, Singapore, 117543 Singapore

## Abstract

The Hippo pathway is a tumor suppressor pathway that is implicated in the regulation of organ size. The pathway has three components: the upstream regulatory factors, the kinase core, and the downstream transcriptional machinery, which consists of YAP, TAZ (transcription co-activators) and TEAD (transcription factor). Formation of YAP/TAZ-TEAD complexes leads to the transcription of growth-promoting genes. Herein, we report the crystal structure of TAZ-TEAD4 complex, which reveals two binding modes. The first is similar to the published YAP-TEAD structure. The second is a unique binding mode, whereby two molecules of TAZ bind to and bridge two molecules of TEAD4. We validated the latter using cross-linking and multi-angle light scattering. Using siRNA, we showed that TAZ knockdown leads to a decrease in TEAD4 dimerization. Lastly, results from luciferase assays, using YAP/TAZ transfected or knockdown cells, give support to the non-redundancy of YAP/TAZ co-activators in regulating gene expression in the Hippo pathway.

## Introduction

The Hippo signaling pathway and its components were first identified and discovered through genetic screens in the fruit fly *Drosophila melanogaster*. It has now been established that the major function of the Hippo pathway is the control of cell number and organ size during early development and for maintaining homeostasis in adulthood^1, 2^. At the molecular level, the regulation of organ size is achieved through the Hippo pathway by regulating cell contact inhibition, cell proliferation and promoting apoptosis^3^. When this delicate control of organ size is dysregulated, cell proliferation goes unchecked and massive outgrowth of tissue occurs. This phenomenon of uncontrolled cell division is one of the hallmarks of cancer^4^. Hence, the Hippo pathway, when it goes awry, is linked to cancer development.

Mammalian homologues of the proteins in the Hippo pathway have been characterized and grouped into three components: the upstream regulatory factors involved in cell-to-cell signaling (FRMD6, NF2), the kinase core (MST, WW45, LATS, MOB1), and the downstream transcriptional machinery (YAP, TAZ, TEAD), which leads to the expression of genes involved in cell proliferation and anti-apoptosis. The kinases in the pathway are considered tumour suppressors and they act by phosphorylating YAP and TAZ, resulting in YAP/TAZ being sequestered in the cytoplasm and consequently promoting their turnover. Being unable to enter the nucleus upon activation of the core kinases, YAP and TAZ, both of which are transcription co-activators, are not able to bind to the transcription factor TEAD to promote transcription of genes involved in cell proliferation and anti-apoptosis^5, 6^.

As potent growth promoters, the overexpression of YAP and TAZ has been shown to promote cell proliferation, migration, invasion, epithelial-mesenchymal transition (EMT), tumourigenesis and is linked to poor prognosis and decreased patient survival^7–9^. In addition, they play a key role in the regulation of stem cell self-renewal and may be a critical link between stem cells and cancer cells^10^. Recent studies have shown that YAP and TAZ can also render cancer cells resistant to common therapeutics, such as taxol and gefitinib^11–13^. More importantly, several groups have provided evidence for the crosstalk between Hippo and other signaling pathways, such as MAPK, Wnt/β-Catenin, and TGF-β pathways; thereby implicating YAP and TAZ as central effectors of several converging pathways^14–18^.

Consequently, YAP and TAZ are candidate oncogenes, and their expression levels and/or increased nuclear accumulation are elevated in several human cancers, including human hepatocellular carcinoma and breast cancer^19–21^. Formation of the YAP/TAZ-TEAD complex within the nucleus leads to transcription of growth-promoting and pro-survival genes, thus promoting malignancy^22^. Conversely, disrupting the interaction between YAP/TAZ and TEAD, or the knockdown of YAP and TAZ, decreases cell proliferation and the oncogenic transforming ability of cells^8, 23, 24^. Small molecule inhibitors and peptides have been identified and are being developed to target the YAP/TAZ-TEAD complex^25, 26^. Some of the small molecule inhibitors were found to bind in a central hydrophobic pocket, where TEAD is reported to be palmitoylated. This posttranslational modification is believed to stabilize and regulate the function of TEAD^27, 28^.

Although YAP and TAZ share a protein sequence similarity of about 42%, are regulated by the same mechanism of the Hippo pathway and bind to similar transcription factors, such as TEAD, several reports suggest that YAP and TAZ can be independently regulated, are non-redundant and have different biological functions due to different transcriptional targets^24^. YAP knockout in mice results in developmental defects and are embryonically lethal, while TAZ knockout results in developmental defects of the kidney and lung^29, 30^. The different functional roles of YAP and TAZ might be due to their ability to promote expression of different target genes, in addition to their differential expressions. In addition, a recent study has shown that the overexpression of YAP negatively regulates TAZ, while YAP knockdown results in increased expression of TAZ. Conversely, TAZ expression levels do not modulate YAP abundance, making it a uni-directional relationship between YAP and TAZ^31^.

The crystal structures of both human and mouse YAP-TEAD complex have been solved previously^32, 33^. The structures reveal that the N-terminal of YAP adopts a helix-loop-helix structure, with helix α1 and helix α2 forming the main hydrophobic and hydrogen-bond interactions with TEAD. The loop of YAP is relatively long and is shown to form four interactions with TEAD as well. Both crystal structures, solved separately by different groups, show one molecule of YAP binding to one molecule of TEAD (1:1 complex). One of the major structural differences between YAP and TAZ is that YAP has a relatively long loop (19 residues) between helix α1 and α2, while TAZ has a shorter loop of 13 residues. Sudol *et al*.^34^ proposed that the lack of a PXXΦP motif in the TAZ linker sequence could result in a unique conformation where each TEAD molecule can interact with two TAZ molecules. In order to ascertain if TAZ and YAP binds to TEAD in the same manner and to shed light on the functional non-redundancy of the two proteins, we set out to solve the structure of TAZ-TEAD complex.

Herein, we report the crystal structure of mouse TAZ-TEAD4 complex, which reveals a distinct binding mode not observed in YAP-TEAD complex. Validation by *in vitro* crosslinking, multi-angle light scattering and immunoprecipitation shows that TAZ-TEAD can form a heterotetrameric complex, with two molecules of TAZ binding to two molecules of TEAD4. Coupled with results from cell-based luciferase assays, using YAP/TAZ transfected or knockdown cells, our structure of TAZ-TEAD complex provides support for the functional non-redundancy of YAP/TAZ co-activators, in regulating gene expression in the Hippo pathway.

## Results and Discussion

### Overall structure

**Figure 1:**
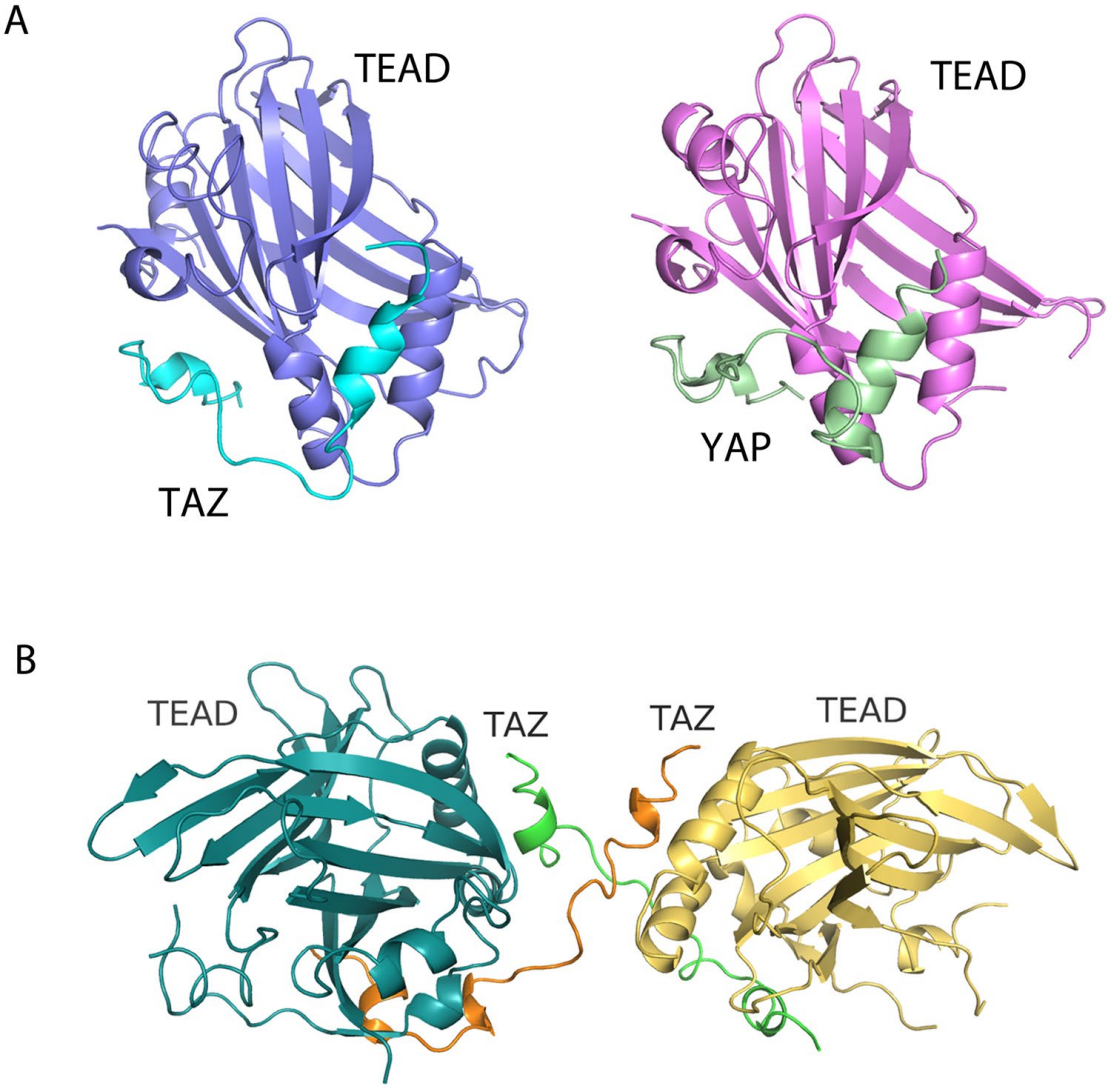
Overall structure of TAZ-TEAD complex. (**A**) Comparison of mTAZ-TEAD4 and previously solved mYAP-TEAD4 (PDB: 3JUA)^32^ complex structures reveals different position of the loop between helix α1 and helix α2. (**B**) A unique binding mode is observed in the crystal structure: two TAZ molecules straddling two TEAD molecules. The TEAD molecules are related by a two-fold symmetry and two TAZ molecules criss-cross each other to bring two TEAD molecules together.

We have solved the crystal structure of the mouse TAZ-TEAD4 complex to a resolution of 2.9 Å. Data collection and refinement statistics are shown in Table 1. The TAZ-TEAD complex crystallizes with four molecules of TAZ and four molecules of TEAD in the asymmetric unit. All four molecules of TEAD adopt a globular structure similar to that seen in the previously solved YAP-TEAD structures. It consists of a central β-sandwich fold and four α-helices on one side. The structures of all four molecules of TAZ reveal two short helices with a short loop of 13 residues in between. The loop lacks the PXXΦP motif that is present in YAP. Further analysis reveals two different binding modes of the TAZ-TEAD complex within the asymmetric unit. In the first binding mode, one TAZ molecule binds to one TEAD molecule to form a heterodimer in a manner similar to that observed in the structures of YAP-TEAD complex^32, 33^. The binding interfaces for helix α1 and helix α2 of TAZ and YAP to TEAD are virtually identical. The only difference lies in the position of the loop between helix α1 and helix α2. As the loop of TAZ is shorter than that of YAP, it adopts a downward position, while that of YAP is in an upward position (Fig. 1A).

**Table 1:** Data collection and refinement statistics for TAZ-TEAD complex.

	TAZ-TEAD
Unit cell dimensions: a, b, c, α, β, γ (Å, °)	79.7, 120.9, 196.6, 90.0, 90.0, 90.0
Space group	P2_1_2_1_2_1_
Beamline/Detector	BL13C1/ADSC Q210
Molecules per asu	4 TAZ, 4 TEAD
Resolution range (Å)	30–2.9
No. of unique reflections	42312 (5796)
Completeness (%)	98.6 (94.5)
Multiplicity	6.2 (5.8)
R_sym_ (%)	16.0 (37.6)
I/σ (I)	8.3 (4.2)
R_work_/R_free_ (%)	22.14/29.11
No. of waters	165
r.m.s.d.^1^ in bond length (Å)	0.013
r.m.s.d. in bond angle (°)	1.75

^1^r.m.s.d. is the root-mean-square deviation from ideal geometry.

In the second binding mode, we observe two TAZ molecules straddling two TEAD molecules in a heterotetramer conformation. The two TEAD molecules are related by a two-fold symmetry and the two TAZ molecules criss-cross each other to bring the two TEAD molecules together (Fig. 1B). This conformation is achieved by the binding of TAZ helix α1 to one TEAD molecule and the binding of helix α2 of the same TAZ to a second TEAD. The loop between the two helices is stretched between the two TEAD molecules. This heterotetramer structure represents a distinct binding mode that has not been observed in the highly-related YAP-TEAD complex.

### Interactions between TAZ and TEAD

**Figure 2:**
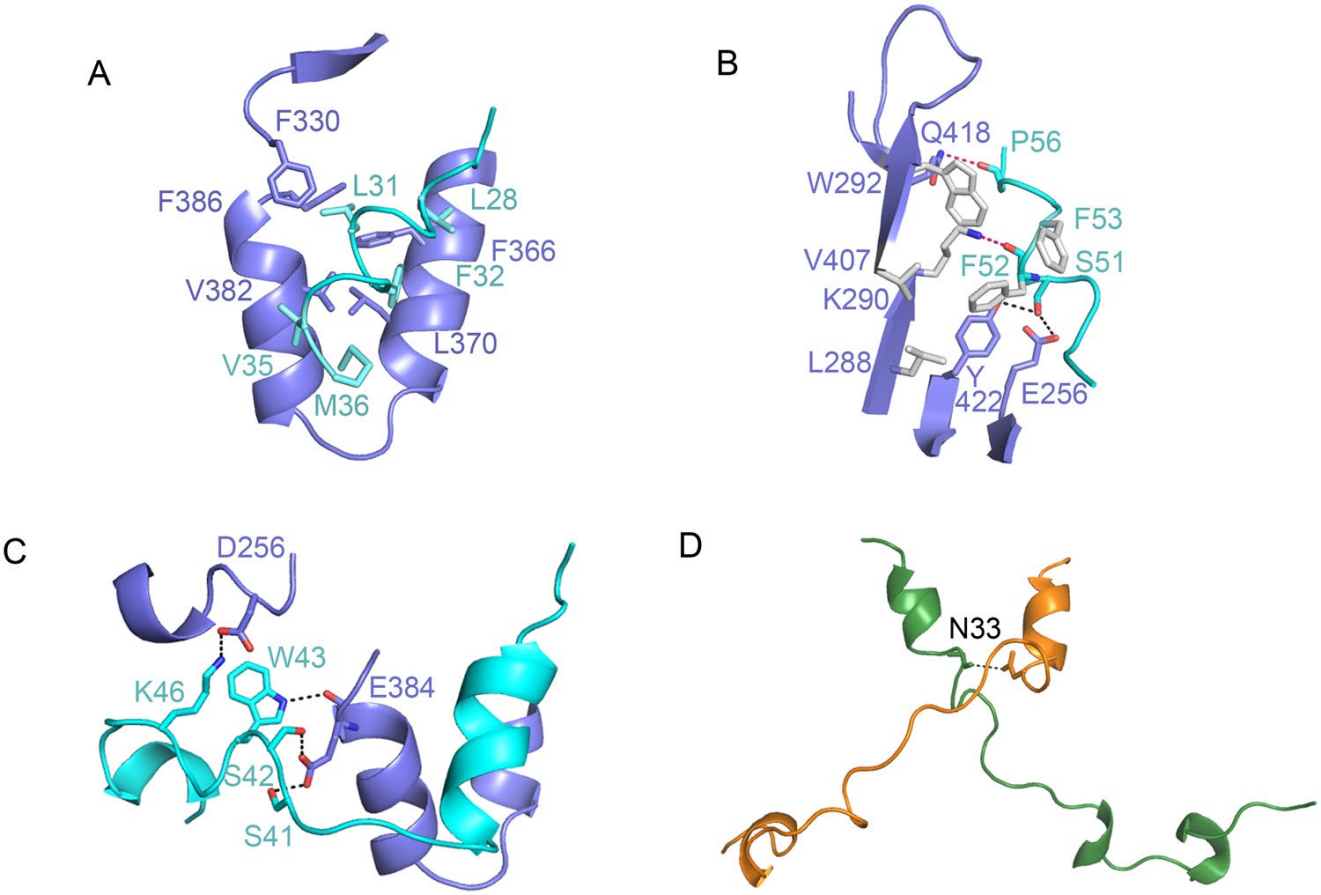
Interactions between residues at the TAZ-TEAD interface. (**A**) The interface between TAZ helix α1 and TEAD reveals a hydrophobic patch of TAZ helix α1 (cyan) interacting with a hydrophobic groove formed by helix α3, α4 and β6-β7 loop of TEAD (purple). The side chains of residues involved in the interactions are shown in stick representation. (**B**) The second interface, between TAZ helix α2 and TEAD, consists of hydrophobic (grey), Van der Waals’ (red dash) and hydrogen bond (black dash) interactions. (**C**) In the first binding mode, where one TAZ molecule binds to one TEAD molecule, the residues in the loop region of TAZ form hydrogen bonds with TEAD. (**D**) In the second binding mode, where one TAZ molecule binds two TEAD molecules, the loops of two TAZ molecules (green and orange) cross each other and a unique hydrogen bond is formed between the TAZ molecules.

Given that the TAZ-TEAD complex can adopt two different binding modes, we proceeded to look at the interaction between the TAZ and TEAD molecules to identify any differences that may give rise to the different binding modes. There are two main interaction interfaces: the first is between TAZ helix α1 and TEAD, while the second is between TAZ helix α2 and TEAD. In comparing the two binding modes, we found that the interface between TAZ helix α1 and TEAD is virtually identical for both binding modes: a hydrophobic patch of TAZ helix α1 interacts with a hydrophobic groove formed by helix α3, α4 and β6-β7 loop of TEAD. The residues involved in this hydrophobic interface are Leu28, Leu31, Phe32, Val35, Met36 of TAZ and Tyr362, Phe366, Lys369, Leu370, Leu373, Met378, Val382, Phe386 of TEAD (Fig. 2A).

In the second interface between TAZ helix α2 and TEAD, we observed hydrophobic, Van der Waals’ and hydrogen bond interactions between residues. These interactions are consistent between the two binding modes. The hydrophobic interactions are formed between residues Phe52, Phe53 of TAZ and the hydrophobic groove formed by Leu288, Lys290, Trp292 and Val407 of TEAD. Van der Waals’ forces are observed between Phe52-Lys290 and Pro56-Gln418 at the TAZ-TEAD interface. Hydrogen bonds were also observed between Ser51 of TAZ and Tyr422 and Glu256 of TEAD (Fig. 2B).

Beside these two main interfaces, a few interactions were also observed between the loop region of TAZ and TEAD. In the first binding mode, where one TAZ molecule binds to one TEAD molecule, the loop region is relatively short and appears to be flexible. Hydrogen bonds are observed between the side chains of Ser41, Ser42, Trp43 of TAZ and the side chain and main chain of Glu384 of TEAD. Lys46 of TAZ also forms a hydrogen bond with Asp265 of TEAD (Fig. 2C). In the second binding mode, where one TAZ molecule straddles two TEAD molecules, only one hydrogen bond interaction is observed between the loop of TAZ and TEAD (Trp43-Glu384), as the loop region stretches between two TEAD molecules and is not in close proximity to TEAD to form many interactions. Instead, the loops of the two TAZ molecules cross each other in this conformation and a hydrogen bond is observed between Asn33 of each of the TAZ molecules (Fig. 2D).

According to Chan *et al*.^8^, the following pairs of residues of TAZ, when mutated to Alanines, resulted in reduced ability to bind with TEAD: Asp27-Leu28, Leu31-Phe32, Trp43-Arg44, Leu48-Phe49, and Phe52-Phe53. From our TAZ-TEAD crystal structure, we show that Leu28, Leu31, Phe32, Leu48, Phe52, and Phe53 of TAZ are important in forming hydrophobic interactions with TEAD. Trp43 and Phe52 of TAZ are also essential in forming Van der Waals’ interactions with TEAD (Fig. 2A–C). These residues and interactions are of great significance because abrogation of these interactions results in the loss of the transforming ability of cells. Point mutations of TEAD4 residues W292, K290 and Y422 (Fig. 2B) were also shown to cause a great reduction in YAP binding and transformation ability^32^. Most significantly, the Y422 residue of mouse TEAD4 is equivalent to Y421 of human TEAD1 and the Y421H missense mutation is the underlying cause of Sveinsson’s chorioretinal atrophy disease in humans^35^. Our TAZ-TEAD structure shows that this tyrosine residue forms a crucial hydrogen bond with Ser51 of TAZ. Hence, our structure provides additional structural basis for the cause of Sveinsson’s chorioretinal atrophy disease.

### Comparison of TAZ-TEAD and YAP-TEAD structures

The comparison of our TAZ-TEAD structure with the two previously published YAP-TEAD structures reveals many similarities and some differences. Several important residues involved in the interaction of the two complexes are highly conserved between TAZ and YAP. Thus, not surprisingly, the hydrophobic interactions at the interface between TAZ/YAP helix α1 and TEAD are virtually identical. The myriad of hydrophobic, Van der Waals’ and hydrogen bond interactions at the interface between TAZ/YAP helix α2 and TEAD are also highly similar. For the first binding mode, where one TAZ molecule binds to one TEAD molecule, the total buried accessible surface area of the three major contact areas (helix α1, loop, helix α2) averages to approximately 1326 Å^2, 36^. This is similar to that observed for the YAP-TEAD structure (~1300 Å)^32^.

**Figure 3:**
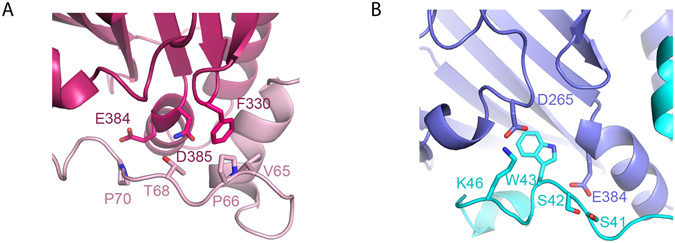
Comparison of YAP-TEAD and TAZ-TEAD structures. (**A**) Interactions between residues on the loop of YAP (pink) and TEAD (red), differ from that of the (**B**) residues on the shorter loop of TAZ (cyan) and TEAD (purple).

The only difference observed between the TAZ-TEAD and YAP-TEAD complexes lies in the loop region. The loop of YAP displays the following interactions with TEAD: Val65-Phe330, Pro66-Phe330, Thr68-Asn385 and Pro70-Glu384 (Fig. 3A). These residues found in the PXXΦP motif of YAP are missing in the shorter loop of TAZ. Hence, in the TAZ-TEAD structure, we observed a different set of interactions: between Ser41, Ser42, Trp43, Lys46 of TAZ and Asp265, Glu384 of TEAD (Fig. 3B). Moreover, in the second binding mode of the TAZ-TEAD structure, we observed fewer interactions than that of YAP-TEAD, as the loop of TAZ extends across two TEAD molecules but is not in close contact to TEAD to form interactions.

Mutagenesis studies done by Chen *et al*.^32^ revealed that the interactions between the PXXΦP motif in the loop of YAP and TEAD is essential for the transforming activity of cells, as a mutated loop (PXXΦP mutated to AXXAA) or deleted loop (loss of PXXΦP motif) resulted in significantly reduced or completely loss of ability of cells to grow in soft agar. However, TAZ, which has a shorter loop that does not consist of a PXXΦP motif, still possess transforming ability. From our TAZ-TEAD structure, we show that the lack of a PXXΦP motif does not affect TAZ binding to TEAD, as other residues (Ser41, Ser42, Trp42, Lys46) of the TAZ loop are able to maintain interactions with TEAD. Hence, a possible explanation for this discrepancy would be that the maintenance of the helix-loop-helix conformation of YAP/TAZ – not the PXXΦP motif – is essential for TEAD binding and consequently the transforming ability of cells^37^.

### TAZ-TEAD forms a heterotetramer *in vitro*

**Figure 4:**
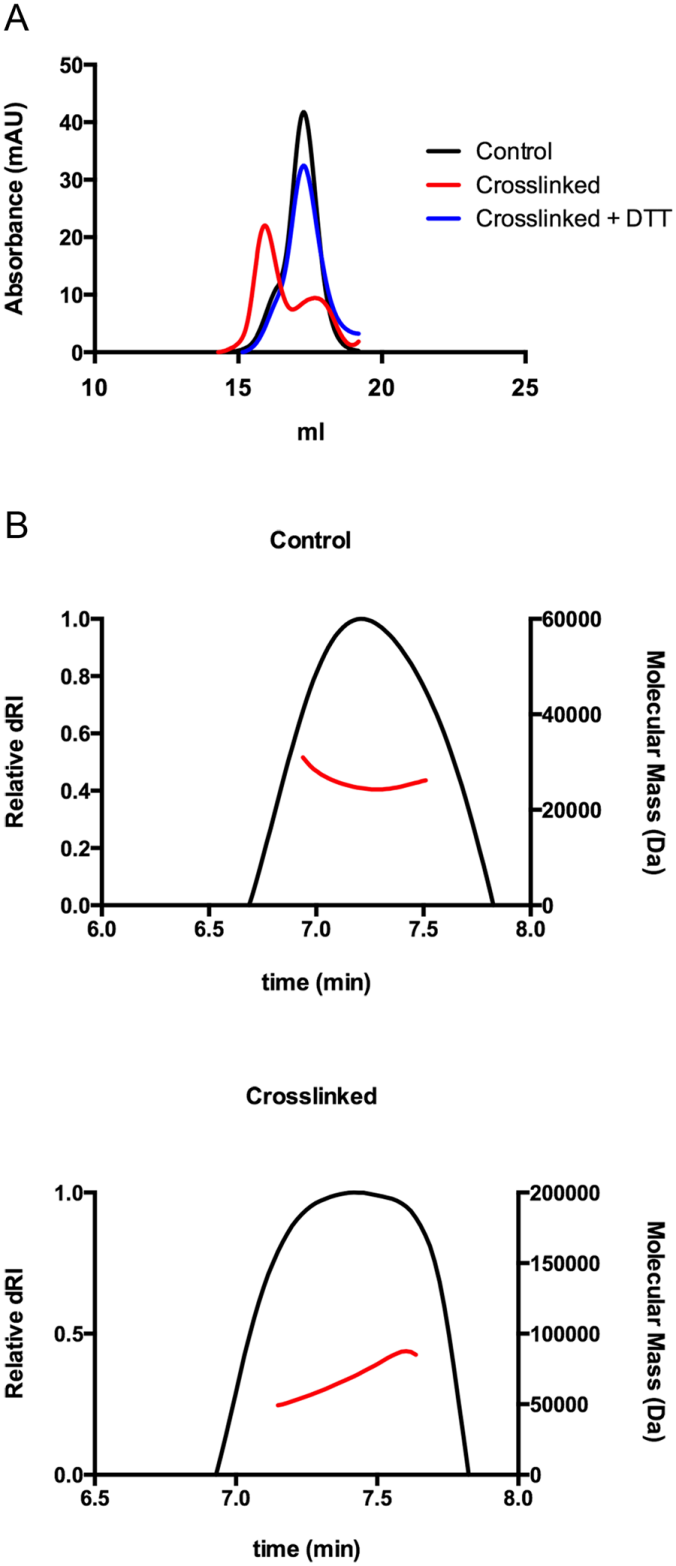
Specific Crosslinking and multi-angle light scattering analysis of TAZ-TEAD complex. (**A**) Analytical gel filtration shows that crosslinking (red) causes the TAZ-TEAD complex to elute earlier than control (without crosslinking, black), indicating an increase in molecular weight. Reversal of crosslinking by addition of DTT (blue) reverts the size of the protein complex to that of the control. (**B**) Differential refractive index of the control (top) and crosslinked (bottom) protein complexes plotted as a function of time. The weight average molecular weight of the protein complex, calculated from light-scattering measurements, is shown as a red continuous line.

In order to validate the distinct binding mode of TAZ-TEAD, we did specific crosslinking of the protein complex and determined the molecular weight of the complex using multi-angle light scattering. Since Asn33 of TAZ is the only residue that is in close proximity (~7.4 Å between Cα) to each other in the heterotetramer conformation, we mutated the residue to cysteine. We also mutated a surface-exposed cysteine (Cys360) of TEAD to serine and proceeded to do specific disulfide crosslinking of the Asn33Cys residue using copper phenanthroline. From the results, we observed that upon specific crosslinking, the proteins form a complex of about 65.0 kD (±1.6%), which is approximately double the size of the protein complex without crosslinking (27.7 kD ± 6.9%). This means that upon specific crosslinking, the proteins likely adopt a heterotetramer conformation of two TAZ molecules binding to two TEAD molecules. Reversing the crosslinking, using the reducing agent DTT, reverts the protein complex to a heterodimer conformation where one TAZ binds to one TEAD, as shown by the analytical gel filtration elution profile (Fig. 4). Thus, the TAZ-TEAD complex probably exists in a dynamic equilibrium between the heterodimer and heterotetramer states in solution.

The TAZ-TEAD heterotetramer conformation observed in our crystal structure is further supported by cell-based data published by Muakami *et al*.^38^. Using co-immunoprecipitation, TAZ, but not YAP, was shown to adopt a homodimer conformation in cells, with the coiled-coil domain of TAZ being important for homodimer formation. Although the longer construct of TAZ, including the coiled-coil domain, did not co-crystallize with TEAD in our hands, the published cell-based data suggests that the coiled-coil domain can precipitate TAZ homodimer formation, which can lead to TAZ-TEAD adopting a heterotetramer conformation. Published results from Muakami *et al*.^38^ might also explain why the two YAP-TEAD crystal structures, solved separately by two groups, did not reveal the possibility of YAP forming a homodimer to bring two TEAD molecules together.

Most recently, a group published the crystal structure of mouse Vgll4-TEAD4 protein complex, which revealed a unique conformation whereby one Vgll4 molecule interacts with two TEAD4 molecules^39^. Although the primary sequence of the TEAD-interacting domain of Vgll differs greatly from that of YAP/TAZ, the first published structure of mouse Vgll1-TEAD4 complex revealed similar binding mode as that of YAP-TEAD: one molecule of Vgll1 binds to one molecule of TEAD via the same interface – helix α1 and hydrophobic groove – of the YAP-TEAD structure^40^. Since the Vgll1-TEAD structure was so similar to that of the YAP-TEAD structure, it came as a surprise that the Vgll4-TEAD structure adopts a different conformation. The major difference between the two homologues is that human Vgll1 has a highly conserved TEAD-interacting motif at its N-terminal (residues 27–51), while human Vgll4 has two partially-conserved TEAD-interacting motifs at its C-terminal (residues 208–219 and 236–247). Hence, in the Vgll4-TEAD structure, one TEAD-interacting motif of Vgll4 binds to one TEAD molecule and the second motif binds to a second TEAD molecule. The linker between the two motifs stretches between two TEAD molecules and brings them close together, but not close enough for any interactions to form between the TEAD molecules. Therefore, the ability of Vgll4 to adopt this unique conformation lends support to our TAZ-TEAD heterotetramer structure.

### Functional non-redundancy of YAP and TAZ

**Figure 5:**
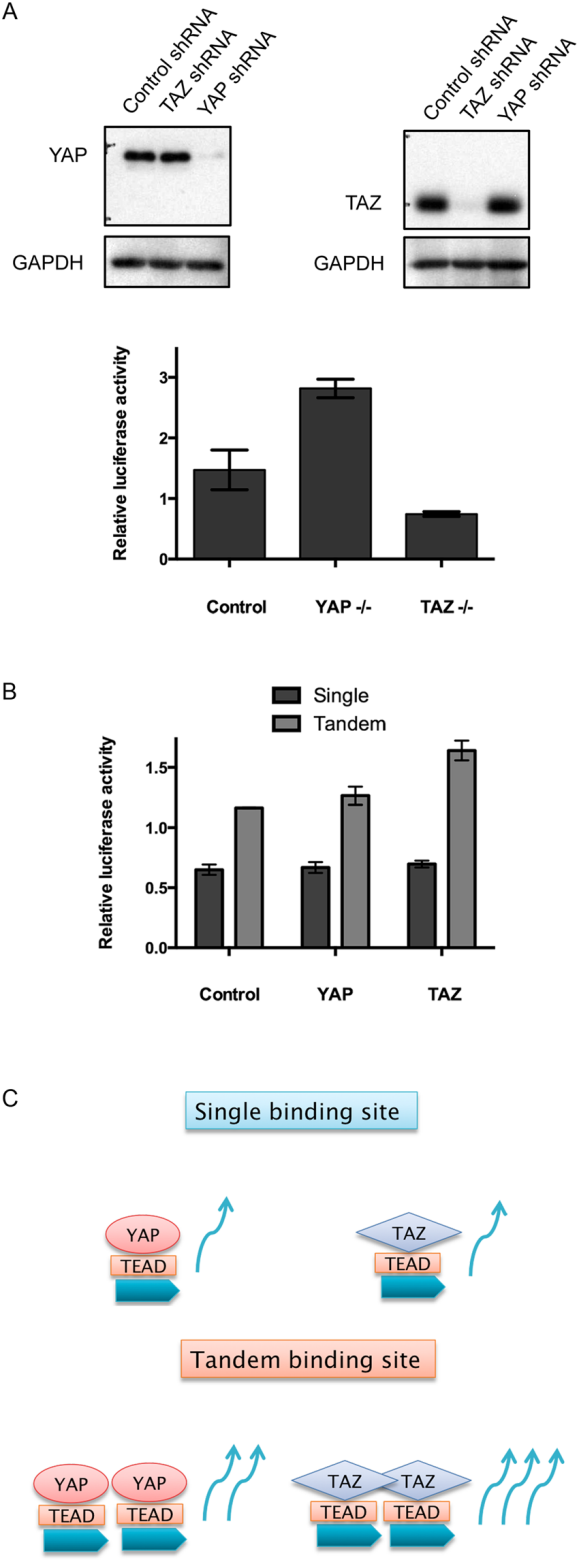
Functional non-redundancy of YAP and TAZ revealed by cell-based luciferase assay. (**A**) YAP knockdown cells show higher reporter activity than TAZ knockdown cells, in the presence of a synthetic 8xGTIIC TEAD luciferase promoter. YAP or TAZ knock down efficiency was checked with antibodies and GAPDH was used as loading control. (**B**) In the presence of a single TEAD binding site at the enhancer, control, YAP and TAZ transfected cells showed similar reporter activity. In the presence of tandem TEAD binding sites, YAP transfected cells showed double the reporter activity (additive), while TAZ transfected cells showed more than double the reporter activity. Each bar represents the mean and standard deviation of three independent experiments. (**C**) Proposed model of how the formation of a TAZ-TEAD heterotetramer complex may result in a stronger additive effect in the presence of tandem TEAD binding sites (blue block arrows).

Having established that TAZ-TEAD can adopt a different conformation from YAP-TEAD complex, we then carried out cell-based luciferase assays to give support to the functional non-redundancy of YAP and TAZ. By knocking down YAP or TAZ expression in H1299 cells, we are able to probe the importance of the individual co-activator TAZ or YAP in promoting transcription by TEAD. It is important to note that when one of the co-activators is knocked down, the expression level of the other co-activator does not increase to compensate for the knock down, but remains the same as that of control cells (Fig. 5A). Nonetheless, the results revealed that cells with TAZ alone not only showed higher reporter activity than cells with YAP alone, but also when compared to control cells (Fig. 5A). This suggests that TAZ possesses stronger co-activator ability, than YAP, to activate TEAD transcription in the presence of the 8xGTIIC promoter^41^. Alternatively, the knock down of YAP could have relieved the repression on TAZ, resulting in significantly higher reporter activity in YAP knock down cells as compared to control cells. Therefore, YAP and TAZ are not only non-redundant in cells, but a knockdown of individual co-activator results in a different outcome, with respect to the promotion of transcription by TEAD.

In order to explain the structure-function relationship of our TAZ-TEAD structure, we performed luciferase assay using YAP or TAZ transfected cells, in the presence of an enhancer containing single or tandem TEAD binding sites. The results showed that in the presence of single TEAD binding site at the enhancer, both YAP and TAZ transfected cells gave rise to similar reporter activity (Fig. 5B). When the enhancer contained a tandem TEAD binding site, YAP transfected cells showed double the reporter activity as compared to single TEAD binding site. This indicates that the binding of two YAP-TEAD heterodimer complexes on the enhancer results in an additive effect. Interestingly, TAZ transfected cells showed more than double the reporter activity in the presence of tandem as compared to single TEAD binding site. The higher reporter activity might be due to the formation of a TAZ-TEAD heterotetramer complex on the tandem TEAD binding site.

Based on previously published results, our TAZ-TEAD crystal structure and our functional cell-based data, we propose that in the presence of single TEAD binding site on DNA, YAP-TEAD and TAZ-TEAD adopt a similar heterodimer conformation. In the presence of tandem TEAD binding sites on DNA, two YAP-TEAD heterodimers can bind to produce an additive effect. On the contrary, TAZ can adopt a criss-cross homodimer conformation to bring two TEAD molecules together to produce an even stronger additive effect (Fig. 5C). Such a conformation could be important for the non-redundancy of YAP and TAZ in their functional roles of promoting differential expression of target genes. An example of how a transcription co-activator can modify the DNA-binding specificity of a transcription factor was revealed by Halder *et al*.^42^ in their study of the Vestigial-Scalloped protein complex (Vg-Sd are Drosophila homologues of mammalian Vgll-TEAD). Specifically, the Vg-Sd complex appears to form a heterotetramer complex on tandem binding sites on DNA. Thus, Vg (co-activator) switches the DNA target preference of Sd (transcription factor) from a single to a tandem binding site. The authors also speculate that a single molecule of Vg could bridge two Sd molecules to increase the binding affinity and stability of the complex to DNA. Their speculation has now been confirmed by the Vgll4-TEAD structure^39^ that shows exactly such a unique conformation that brings two transcription factors together.

### Concluding remarks

Our TAZ-TEAD crystal structure reveals two binding modes (1:1 and 2:2 complexes) of the co-activator with its transcription factor. The structure not only reveals the similar mode of action between YAP and TAZ, but also provides additional support for the non-redundancy of YAP and TAZ transcription co-activators. In particular, our cell-based data shows that the formation of a TAZ-TEAD heterotetramer complex may produce a stronger additive effect for the expression of certain target genes, especially those possessing two adjacent binding sites for the transcription factor. Further experiments will be necessary to ascertain if the YAP-TEAD protein complex can also adopt a different confirmation for the expression of different target genes. Most recently, Lee *et al*. showed that the DNA-binding domain of TEAD adopts a homodimer conformation in the crystal structure^43^. The results allude to TEAD switching between monomer and dimer to regulate DNA target selectivity. Crystal structures of YAP/TAZ-TEAD in complex with their DNA targets are thus needed to provide evidence for their binding conformations in the presence of different DNA targets. More work in this area will eventually allow us to understand how the structures of the YAP-TEAD and TAZ-TEAD complexes can give rise to functional non-redundancy and regulation of YAP/TAZ-dependent gene expression.

## Material and Methods

### Protein expression and purification

mTAZ (residues 25–57) and mTEAD4 (residues 210–427) were cloned into a modified pETDuet-1 vector (Novagen), containing a 3C protease-cleavable His_6_-tag at the N-terminal of mTAZ. The proteins were co-expressed in *E*. *coli* BL21 (DE3) strain by induction with 0.5 mM isopropyl β-D-thiogalactopyranoside at 18 °C overnight. Harvested cells were resuspended in buffer A (20 mM Tris pH 8 and 150 mM sodium chloride) supplemented with 1 mM phenylmethylsulfonyl fluoride, 25 ug/ml DNaseI, and 2 mM magnesium chloride. The TAZ-TEAD complex was purified on TALON Co^2+^ resin equilibrated in buffer A and eluted on a stepwise gradient of 250 mM to 500 mM imidazole. Desalting was carried out to remove imidazole before addition of 3 C protease to cleave off the His_6_-tag at 4 °C overnight. Uncleaved protein and protease were removed by a second affinity chromatography step. The cleaved protein was then subjected to size exclusion chromatography on a Superdex75 gel filtration column using a buffer containing 20 mM Tris pH 8, 150 mM sodium chloride, and 5 mM β-mercaptoethanol. The protein complex was concentrated to 20 mg/ml, frozen in liquid nitrogen and stored at −80 °C. For the crosslinking experiment, TAZ Asn33 and TEAD surface-exposed Cys360 were replaced with cysteine and serine respectively, in the abovementioned TAZ-TEAD construct. The protein complex was expressed and purified as described above.

### Crystallization and structure determination

Large crystals of TAZ-TEAD complex appeared after 3–5 days in hanging drops by mixing 4 μl of protein with 4 μl of reservoir solution containing 6% Polyethylene glycol 10000, 0.05 M magnesium acetate, and 0.1 M tri-sodium citrate pH 5.2 at 20 °C. Micro streak seeding was carried out to produce single crystals. Dehydrating solution (12% Polyethylene glycol 10000, 0.09 M magnesium acetate, 0.12 M tri-sodium citrate pH 5.4A, 10% glycerol) was added slowly to the crystal drop until the total volume of the drop was twice the original. The drop was then equilibrated against air for 30 minutes at room temperature. A block-shaped crystal with dimensions of approximately 0.5 × 0.2 × 0.1 mm was then flash frozen in liquid nitrogen.

The diffraction data for the TAZ-TEAD complex were measured at the National Synchrotron Radiation Research Center (NSRRC), Taiwan on beamline BL13C1. The data was processed using iMosflm^44^ and scaled using Scala^45^ from the CCP4^46^ suite of programs, to a resolution of 2.9 Å. The calculation of R_free_ used 5% of data. The structure of the complex was solved by molecular replacement with Phaser^47^ from the CCP4 suite of programs using the mTEAD4 molecule in the mYAP-mTEAD4 structure as a search model (PDB code: 3JUA)^32^. Initial refinement was carried out with Refmac5^48^ using rigid body refinement followed by restrained refinement. Electron density and difference density maps were inspected and the model was improved using Coot^49^. Further refinement was carried out using Phenix^50^. The position and conformation of TAZ in the second binding mode was confirmed by the generation of a Fo-Fc omit map (Figure S1). Protein domain interfaces were analyzed using PDBePISA^36^. Figures were prepared using PyMOL^51^.

### Specific crosslinking and multi-angle light scattering

Prior to crosslinking, desalting was carried out to remove β-mercaptoethanol from the protein buffer. Specific disulfide crosslinking of the protein (100 μg) was initiated by the addition of 1.5 mM CuCl_2_ and 5 mM phenanthroline for 1 hour at 25 °C. The reaction was stopped by the addition of 50 mM EDTA for 15 minutes at 25 °C. Reversal of crosslinking was carried out by adding 25 mM DTT for 1 hour at 25 °C. The elution profiles of the products were monitored by analytical gel filtration using the Superdex 75 10/300 GL column (GE Healthcare). Analytical size exclusion chromatography and multi-angle light scattering (MALS) was performed using a Superdex 75 5/150 GL column (GE Healthcare), connected upstream of the UV/Vis detector, Wyatt DAWN HELEOS II light scattering detector and Wyatt Optilab refractive index monitor. The system was equilibration with 20 mM Tris pH 8 and 150 mM sodium chloride, before injecting 25 ul of the protein sample (6 mg/ml) into the column. Data analysis was carried out using ASTRA software (Wyatt). Graphs were prepared using Prism 6 (GraphPad).

### Luciferase assay

H1299 cells were stably transduced with control shRNA, YAP-shRNA and TAZ-shRNA^21^. The YAP or TAZ protein knock down efficiency was checked with antibodies (Cell Signaling Technology). Cells were grown in RPMI media supplemented with 10% fetal bovine serum and puromycin for selection. Cells in 24-well plates were transfected with 0.5 ug of synthetic 8xGTIIC TEAD luciferase promoter^41^ and 0.1 ug of Renilla luciferase control reporter, using Lipofectamine2000 (Thermo Fisher Scientific). Luciferase assay was performed 24 hours after transfection using the Dual-Luciferase Reporter Assay system (Promega), according to manufacturer’s guidelines.

Single (TGTGGAATGTGT) and tandem (TGTGGAATGTGTGGAATGTGT) TEAD binding sites were cloned upstream of the SV40 promoter of the pGL3-promoter vector. Non-transduced H1299 cells were transfected with 0.1 ug co-activator (pCI control vector, YAP or TAZ), 2.5 ug of pGL3-promotor (control, single or tandem binding sites), and 0.5 ug of Renilla luciferase reporter, using Lipofectamine2000. Luciferase assay was performed 24 hours after transfection. Graphs were made using Prism 6 and all results shown are of triplicates.

## Electronic supplementary material

Supplementary Information
