# Supplementary material for: Crystal structure of TAZ-TEAD complex reveals a distinct interaction mode from that of YAP-TEAD complex
Source: Sci Rep. 2017 May 17;7:2035. doi: 10.1038/s41598-017-02219-9 (PMC5435683; doi:10.1038/s41598-017-02219-9)
Supplement: Supplementary file 1 — Supplementary Information [file 41598_2017_2219_MOESM1_ESM.pdf]

Supplemental Information

Crystal structure of TAZ-TEAD complex reveals a distinct interaction mode from that of YAP-TEAD complex

Hung Yi Kristal Kaan, Siew Wee Chan, Siew Kim Joyce Tan, Fusheng Guo, Chun Jye Lim, Wanjin Hong, and Haiwei Song

Figure S1

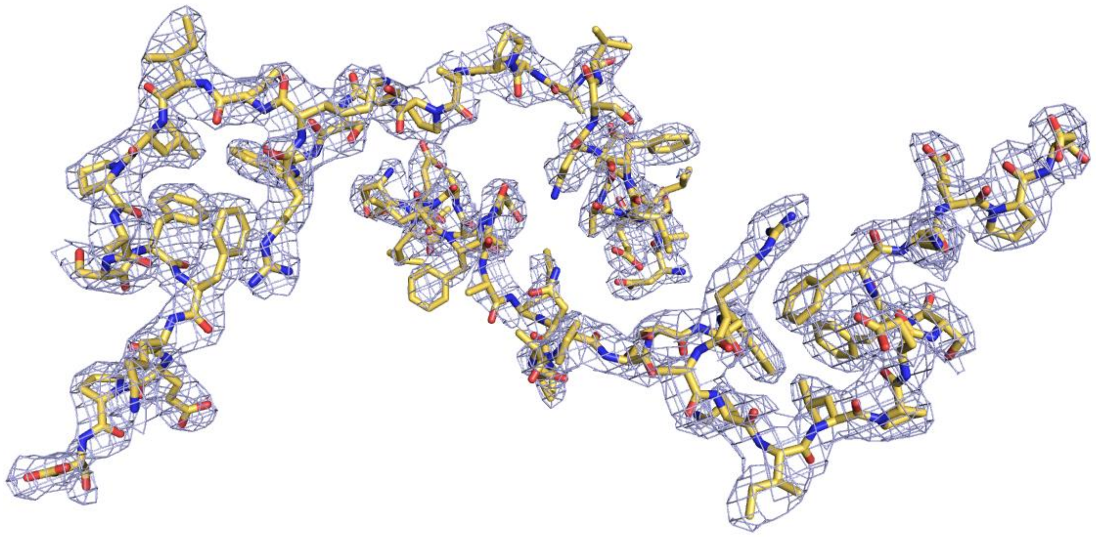

The distinct binding mode of TAZ (yellow sticks) to TEAD is revealed by the generation of a f_o-f_c omit map (contoured at σ=3.00), to visualize the position and conformation adopted by the TAZ molecules.
